# Recent Uses of *N*,*N*-Dimethylformamide and *N*,*N*-Dimethylacetamide as Reagents

**DOI:** 10.3390/molecules23081939

**Published:** 2018-08-03

**Authors:** Jean Le Bras, Jacques Muzart

**Affiliations:** Institut de Chimie Moléculaire de Reims, CNRS—Université de Reims Champagne-Ardenne, B.P. 1039, 51687 Reims CEDEX 2, France; jean.lebras@univ-reims.fr

**Keywords:** *N*,*N*-dimethylformamide, DMF, *N*,*N*-dimethylacetamide, DMAc, amination, amidation, thioamidation, formylation, carbonylation, cyanation, insertion, cyclization

## Abstract

*N*,*N*-Dimethylformamide and *N*,*N*-dimethylacetamide are multipurpose reagents which deliver their own H, C, N and O atoms for the synthesis of a variety of compounds under a number of different experimental conditions. The review mainly highlights the corresponding literature published over the last years.

## 1. Introduction

**Scheme 1:**
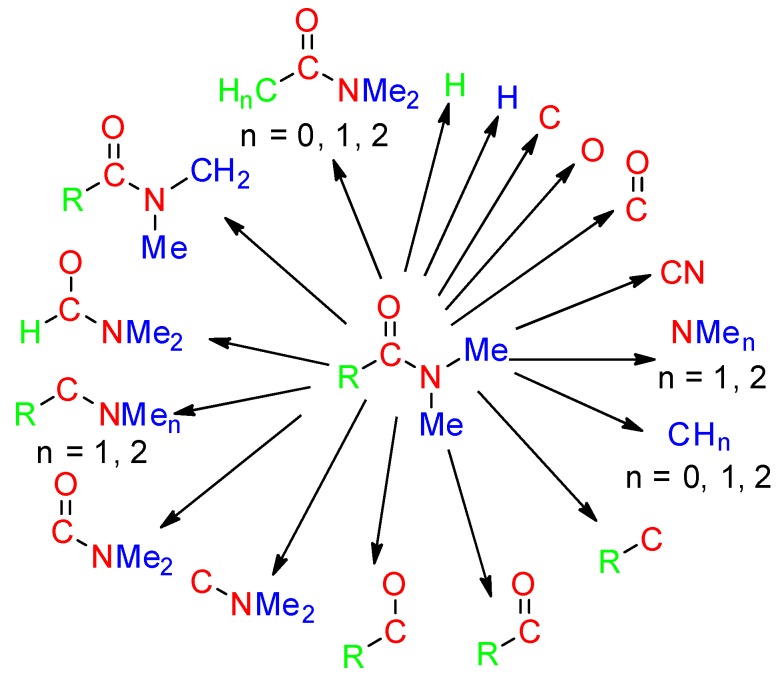
Fragments from DM (R = H or Me) used in synthesis.

The organic, organometallic and bioorganic transformations are extensively carried out in *N*,*N*-dimethylformamide (DMF) or *N*,*N*-dimethylacetamide (DMAc). These two polar solvents are not only use for their dissolution properties, but also as multipurpose reagents. They participate in a number of processes and serve as a source of various building blocks giving one or more of their own atoms (Scheme 1).

**Scheme 2:**
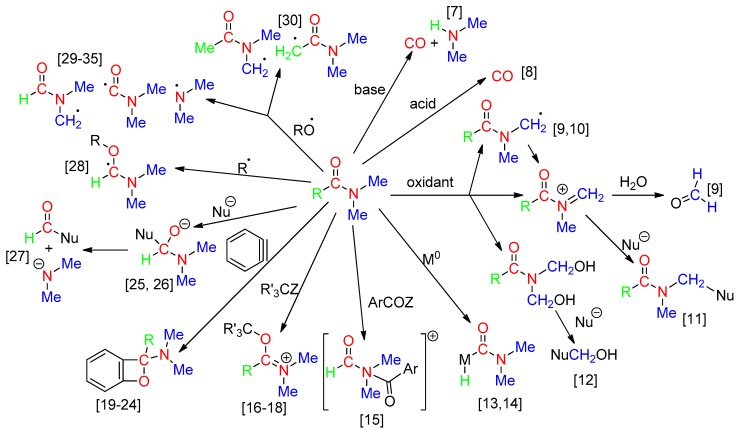
Reactions of DM (R = H or Me).

In 2009, one of us reviewed the different roles of DMF, highlighting that DMF is much more than a solvent [1]. Subsequently, this topic has been documented by the teams of Jiao [2] and Sing [3]. For of a book devoted to solvents as reagents in organic synthesis, we wrote a chapter summarizing the reactions consuming DMF and DMAc as carbon, hydrogen, nitrogen and/or oxygen sources [4]. This book chapter tentatively covered the literature up to middle 2015. The present mini-review focuses on recent reactions which involve DM (DM = DMF or DMAc) as a reagent although some key older papers are also included for context. Processes which necessitate the prerequisite synthesis of DM derivatives such as the Vilsmeier-Haack reagents [5] and DMF dimethyl acetal [6] are not surveyed, but a few reactions of the present review involve the in-situ formation of a Vilsmeier-type intermediate (Vilsmeier-type reagents have been extensively used. Search on 26 June 2018 for “Vilsmeier” with SciFinder led to 4379 entries). Color equations, based on literature proposals, are used to easily visualize the DM atom origin. When uncertainty is expressed by the authors or suspected by us, the atom is typed in italic. Mechanistic schemes are not reported, but Scheme 2 [7,8,9,10,11,12,13,14,15,16,17,18,19,20,21,22,23,24,25,26,27,28,29,30,31,32,33,34,35] summarizes different proposed reactions of DM with the corresponding literature references, where DM acts as either a nucleophilic or electrophilic reagent, or leads to neutral, ionic or radical species. The review is divided in Sections depending on the DM fragment(s) which is (are) incorporated into the reaction product.

## 2. C Fragment

Aerobic carbonylation under nickel/copper or palladium/silver synergistic catalysis occurred efficiently using the Me group of DMF as the C source, affording cyclic carbonylated compounds, via the directing group-assisted activation of a C(sp^2^)–H or C(sp^3^)-H bond (Equations (1) and (2) [36], Equations (3) and (4) [37]). Shifting from DMF to DMAc greatly decreased the yields (Equations (1) and (3)).


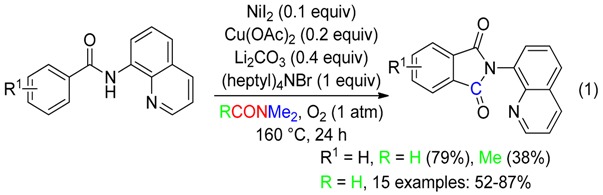



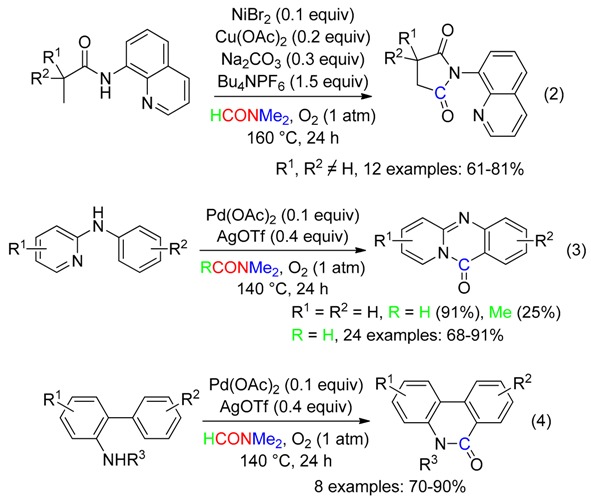


The Me group of DMF was also involved in the cyanation of the C(sp^2^)-H bond of arenes catalyzed with an heterogeneous copper catalyst (Equation (5)) [38].


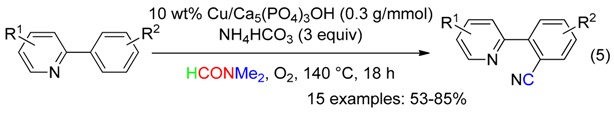


## 3. CH Fragment

Treatment of indole at 130 °C with suprastoichiometric amounts of CuI, *t*-BuOOH and AcOH in DMAc under air afforded the corresponding C3-formylation product (Equation (6)) [39]. Such a reaction also occurred with *N*-methylindole using CuI and CF_3_CO_2_H in DMAc under oxygen [40]. The CH fragment came from the NMe_2_ moiety [39,40]. In DMF, both procedures led to C3-cyanation (see below, Equation (25)).


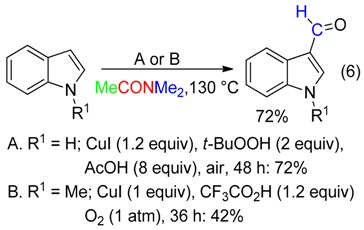


Cycloadditions leading to symmetrical tetrasubstituted pyridines using the Me group of DMF as the CH source have been carried out using either ketoxime carboxylates with Ru catalysis (Equation (7)) [41], or arones with both iodine and ammonium persulfate mediation (Equation (8)) [42].


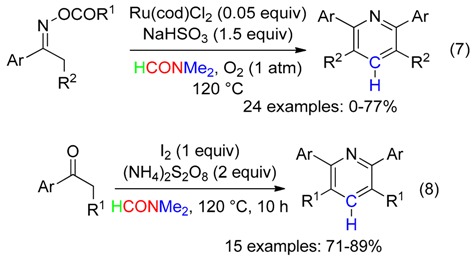


2,4-Diarylpyridines were also synthetized from Ru-catalyzed reaction of acetophenones with ammonium acetate and DMF as source of the N and CH atoms, respectively (Equation (9)) [43].


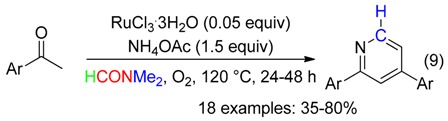


With sodium azide as the nitrogen source, DMAc was superior to DMF to deliver the CH fragment of the copper-catalyzed domino reactions of aryl halides which led to imidazo [1,2-*c*]quinazolines, quinazolinones or imidazo[4,5-*c*]quinolones (Equations (10)–(12)) [44].


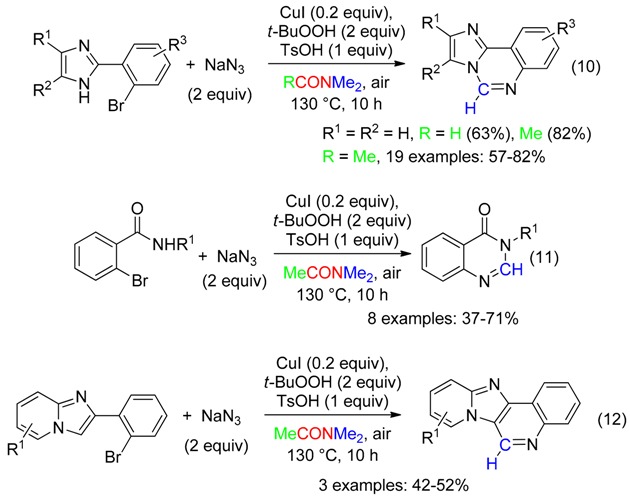


In contrast to the above examples, the Cu-catalyzed cyclization leading to 6*H*-chromeno[4,3-*b*]quinolin-6-ones (Equation (13)) with incorporation of a CH belonging to DMF occurred with low yield when *t*-BuOOH was the oxidant. Shifting to *t*-butyl perbenzoate allowed an effective reaction [45]. For the synthesis of 4-acyl-1,2,3-triazoles from Cu-catalyzed cycloaddition to acetophenones (Equation (14)), K_2_S_2_O_8_ was superior to *t*-BuOOH, (*t*-BuO)_2_ and (PhCO_2_)_2_ [46]. Yields decreased with DMAc instead of DMF (Equations (13) and (14)).


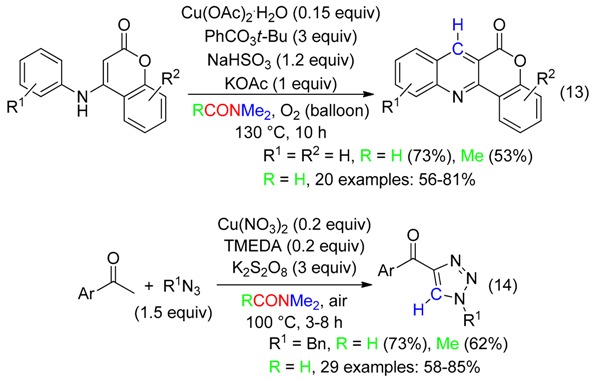


The insertion of a CH from the NMe_2_ of DM under metal-free conditions has been reported for the synthesis of cyclic compounds such as:

- pyrimidines from *t*-BuOOH-mediated reaction between acetophenones, amidines and DMF (Equation (15)) [47],
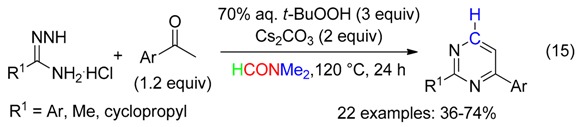
- substituted phenols also from three components cycloadditions (Equation (16)) [48],
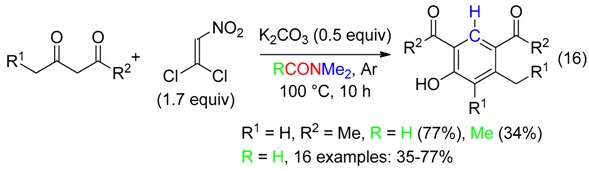
- 3-acylindoles from 2-alkenylanilines (Equation (17)) [49],
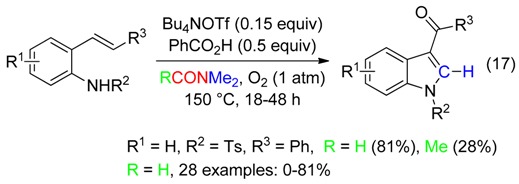
- benzimidazoles and benzothiazole from *o*-phenylenediamine or 2-aminobenzenethiol through carbon dioxide-mediated cyclization (Equation (18)) [50].
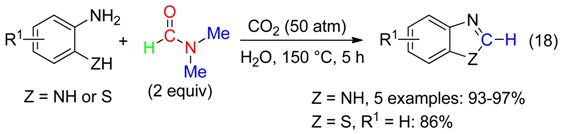

## 4. CH_2_ Fragment

The coupling of indoles or imidazo[1,2-*a*]pyridines to afford heterodiarylmethanes with DMF as the methylenating reagent occurred in fair to high yields with a Cu^I^ catalyst associated to *t*-BuOOH [51] or K_2_S_2_O_8_ [52] (Equations (19)–(21)). Use of DMAc was less efficient.


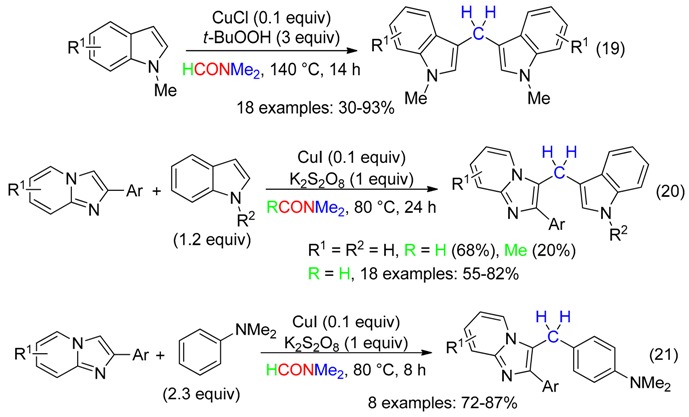


In DMAc, the I_2_/*t*-BuOOH association catalyzed the formation of methylene-bridged bis-1,3-dicarbonyl compounds from aryl β-ketoesters or β-ketoamides (Equation (22)). Lower yields were obtained with I_2_/K_2_S_2_O_8_ in DMAc or DMF [9]. Subjection 1,3-diphenylpropane-1,3-dione or ethyl 3-oxobutanoate to the I_2_/*t*-BuOOH/DMAc did not afford the bridged compounds.


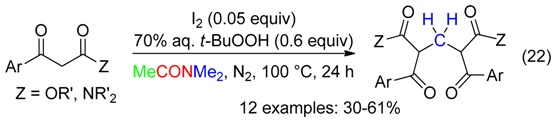


A Mannich reaction leading to β-amino ketones with DMF as the formaldehyde source has been reported in the presence of *t*-BuOOH and catalytic amounts of an *N*-heterocyclic carbene, SnCl_2_ and NEt_3_ (Equation (23)) [11].


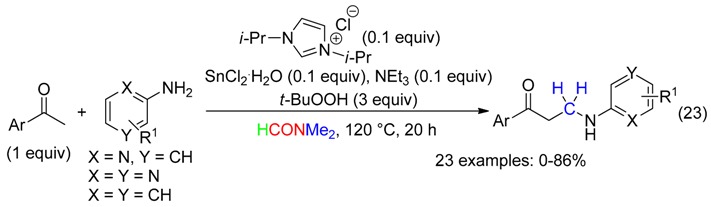


The study of an unexpected reaction due to the oxidation of DMAc with aqueous *t*-BuOOH (Equation (24)) showed the formation of MeCONMe(CH_2_OH) and MeCON(CH_2_OH)_2_, these unusual species delivering the methylene group [12].


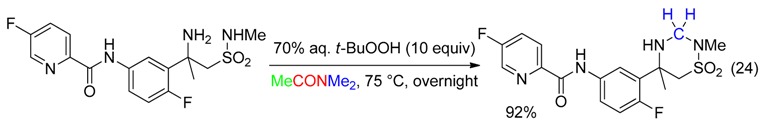


## 5. NC Fragment

While CuI under oxidative and acidic conditions led, in DMAc, to the C3-formylation of indole and *N*-methylindole (Equation (6)), reactions in DMF led to C3-cyanations (Equation (25)) [39,40]. Cyanation of electron-rich arenes and benzaldehydes was also carried out (Equation (26)) [39]. Monitoring the course of the reaction indicated a cyanation arising via the formyl compounds [39,40]. Moreover, 3-iodo indole could also be involved in the formation of the cyano product [40].


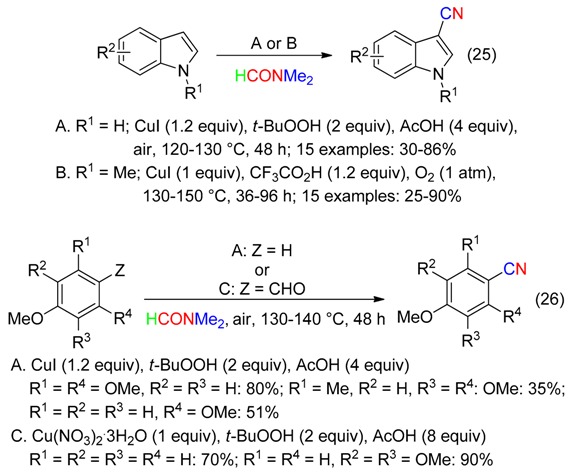


Laser ablation of silver nitrate in DMF led to silver cyanide (Equation (27)) [53].


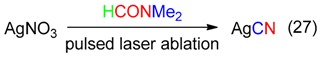


## 6. NMe_2_ Fragment

This chapter is divided in sections corresponding to the type of function reacting with DM.

### 6.1. Aryl Halides

Refluxing chloropyridines in DM or DMAc afforded the corresponding aminopyridines (Equation (28)) [25]. The amination of aryl chlorides and 3-pyridinyl chloride with DMF occurred at room temperature in the presence of potassium *t*-butoxide and a carbenic palladium catalyst (Equation (29)) [27].


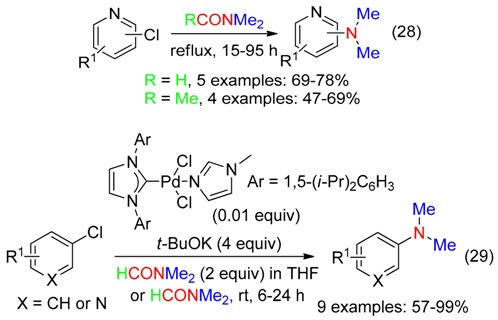


### 6.2. Alkylarenes

Oxidation of methylarenes and ethylarenes at 80 °C in DMF using catalytic amounts of both I_2_ and NaOH [31] or *n*-Bu_4_NI [32] associated to aqueous *t*-BuOOH under air led to benzylic oxidation and incorporation of the NMe_2_ fragment to afford benzamides (Equation (30)) or α-ketoamides (Equation (31)).


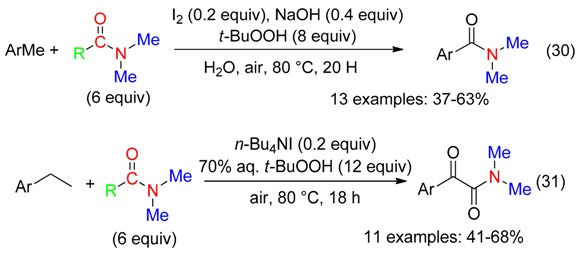


### 6.3. Alkenes

Hydrocarbonylation of terminal alkenes and norbornene followed by acyl metathesis with DM occurred under Pd catalysis, CO pressure and in the presence of ammonium chloride or *N*-methyl-2-pyrrolidone hydrochloride (NMP·HCl) (Equations (32) and (33)) [54]. From alkenes, the selectivity towards linear and branched products depended on the catalytic system (Equation (32)). DMF and DMAc afforded similar results.


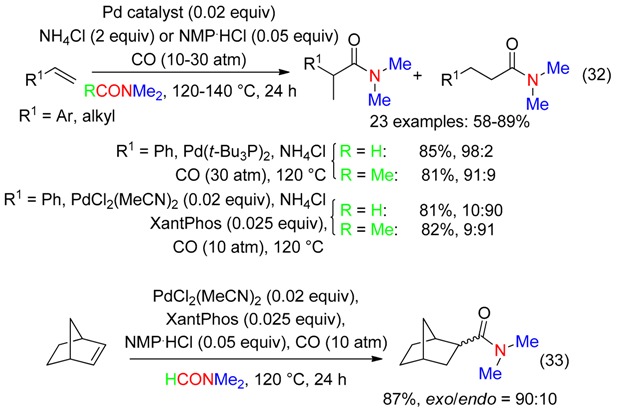


### 6.4. Acids

Copper, palladium and ruthenium catalysts associated to oxidants and DMF were used for the amidation of cinnamic acids [29] and carboxylic acids [55] (Equations (34) and (35)). *N*,*N*-Dimethylbenzamide was one of the products obtained from the Cu^II^-catalyzed oxidation of flavonol [56].


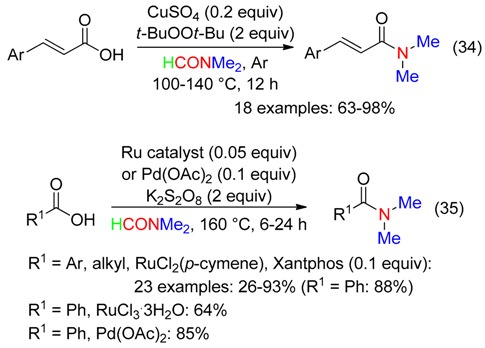


Metal-free conditions and DMF were used for:

- the amidation of acids promoted with propylphosphonic anhydride associated to HCl at 130 °C (Equation (36)) [15],
- the amination of acids employing a hypervalent iodine reagent at room temperature (Equation (37)) [35]. Mesityliodine diacetate was superior to the other hypervalent iodine reagents, while oxidants such as I_2_, *t*-BuOOH, NaIO_4_ or K_2_S_2_O_8_ did not mediate the amidation reaction [35].
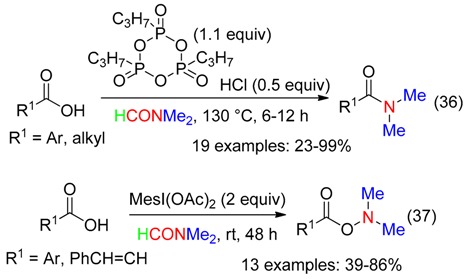

Treatment of arylacetic and cinnamic acids with base, sulfur and DMF at 100–120 °C led to decarboxylative thioamidation (Equations (38) and (39)) [7]. Inhibition of the process in the presence of TEMPO or BHT indicated a radical involvement in the transformations.


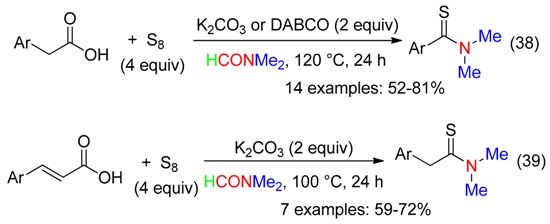


### 6.5. Carbonylated Compounds

Reaction of 2-arylquinazolin-4(3*H*)-ones with TsCl and *t*-BuOK in DM provided the corresponding 4-(dimethylamino)quinazolines in good yields, especially in DMF (Equation (40)). These reactions occurred via the formation of the 2-aryl-4-(tosyloxy)quinazolines [57].


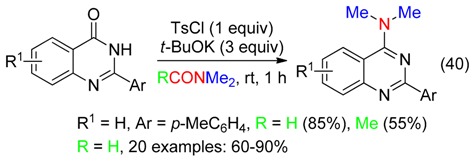


Various amides have been synthetized from aldehydes and DMF using *t*-BuOOH and a recyclable heterogeneous catalyst—a carbon–nitrogen embedded cobalt nanoparticle denoted as Co@C-N600 (Equation (41)) [33]. The same transformation of benzaldehydes was subsequently reported using Co/Al hydrotalcite-derived catalysts [58].


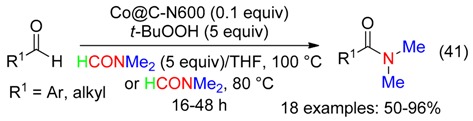


Copper oxide and iodine mediated the reaction of acetophenones with sulfur and DMF to afford α-arylketothioamides (Equation (42)) via the formation of α-iodoacetophenones [59].


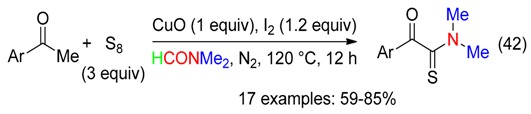


Elemental sulfur and the NMe_2_ moiety of DMF or DMAc was also used for the DBU-promoted synthesis of thioamides from aldehydes (Equation (43)) or arones (Equation (44)) [60], the latter undergoing an efficient Willgerodt-Kindler reaction [61,62].


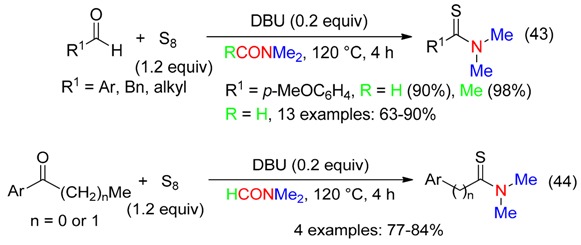


### 6.6. Benzyl Amines

The recyclable Co/Al catalysts used above in DMF for the amidation of benzaldehydes also led to benzamides from benzylamines and *t*-BuOOH (Equation (45)). These transformations would involve benzaldehydes as intermediates [58].


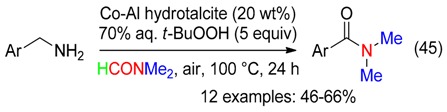


### 6.7. Nitriles

NaOH mediated, at room temperature, the efficient reaction of the CN group of 4-oxo-2,4-diphenylbutanenitrile with DMF to afford the corresponding γ-ketoamide (Equation (46)) [63]. Such compounds were also obtained from the domino reaction of chalcones with malononitrile and NaOH in DMF [63].


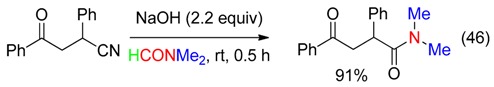


### 6.8. Sulfur Compounds

Sulfonamides were synthetized:

- from thiophenols, DMF and air via an oxygen-activated radical process mediated by copper salts and cinnamic acid (Equation (47)) [64],
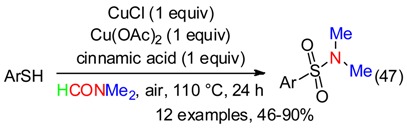
  or reaction of sodium sulfonates with *N*-iodosuccinimide and DMF pretreated with *t*-BuOK (Equation (48)) via, probably, sulfonyl iodides (Equation (49)) [65].
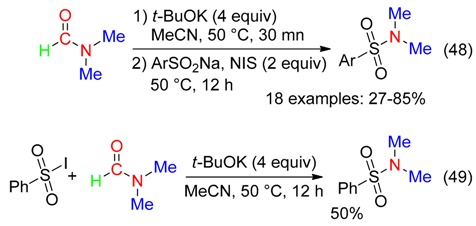

## 7. O Fragment

DMF delivered its oxygen atom to 1,2-cyclic sulfamidates via nucleophilic displacement at the quaternary center to afford, after hydrolysis, an aminoalcohol (Equation (50)) [17].


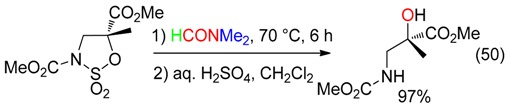


DMF was also the oxygen source leading to an imidazolinone from the reaction with the Cu-carbene complex and the borate salt depicted in Equation (51) [66].


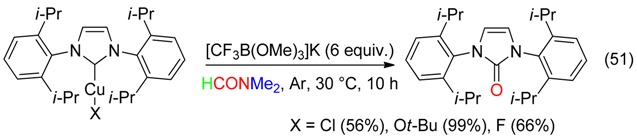


The I_2_/CuO association allowed the α-hydroxylation of arones in abstracting, via the α-iodoarone, the oxygen atom of DMF (Equation (52)) [18].


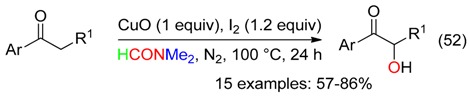


## 8. C=O Fragment

With DMF as the CO surrogate, quinazolinones have been prepared at 140–150 °C

- via C(sp^2^)-H bond activation and annulation using Pd/C [67] or Pd(OAc)_2_ [8], in the presence of K_2_S**_2_**O_8_, CF_3_CO_2_H and O_2_ (Equation (53)),
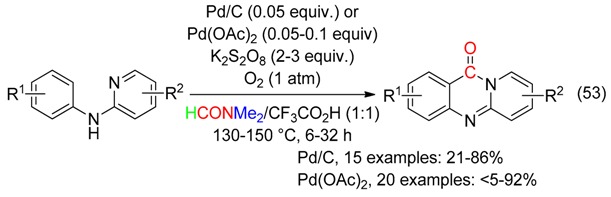
  or carbon dioxide-mediated cyclization of 2-aminobenzonitrile (Equation (54)) [50]. This latter reaction would involve a Vilsmeier-Haack type intermediate and did not occur with DMAc.
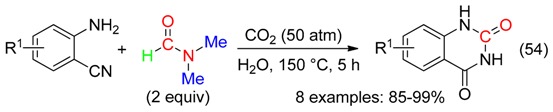

A carbonylative Suzuki-type reaction leading to diarylketones arose in DMF under Ni catalysis at 100 °C (Equation (55)), IPr = bis(2,6-diisopropylphenyl)imidazol-2-ylidene) [13], or Pd catalysis and UV light assistance at room temperature (Equation (56)) [68].


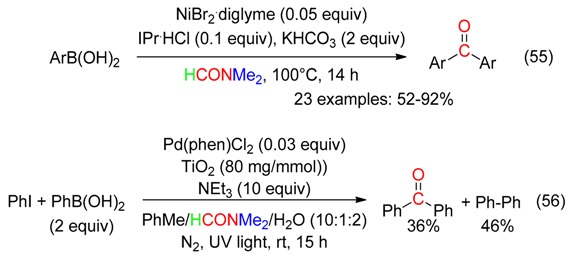


Catalytic amounts of a ruthenium pincer complex and *t*-BuOK led, at 165 °C, to symmetric and unsymmetric *N*,*N*’-disubstituted ureas from primary amines and DMF (Equation (57)) [69].


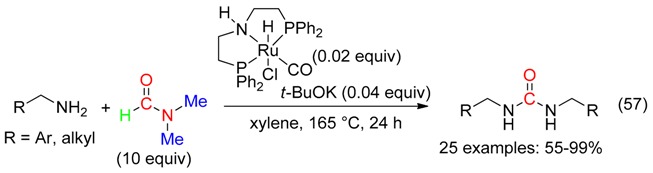


At 120 °C under CuBr_2_ catalysis, *o*-iodoanilines reacted with potassium sulfide and DMF, leading to benzothiazolones (Equation (58)) [70].


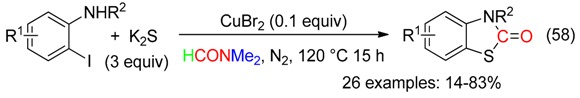


## 9. C=ONMe_2_ Fragment

Potassium persulfate-promoted the reaction of pyridines with DMF to provide *N*,*N*-dimethylpicolinamides (Equation (59)) [71], while the oxidative carbamoylation of isoquinoline *N*-oxides, with also DMF, was catalyzed by Pd^II^ in the presence of ytterbium oxide as base and tetrabutylammonium acetate, the latter mediating the N-O reduction (Equation (60)) [72].


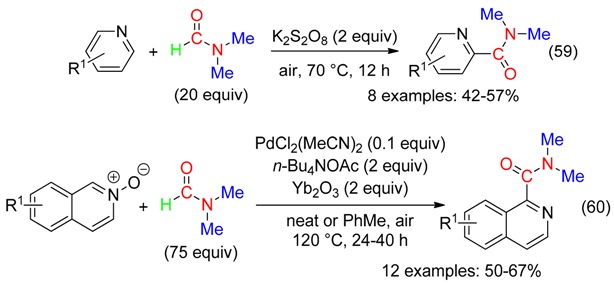


Alkynylation of DMF leading to *N*,*N*-dimethylamides was produced with peroxides and either an hypervalent alkynyl iodide under Ag catalysis (Equation (61)) [73], or a terminal alkyne under Cu catalysis (Equation (62)) [34].


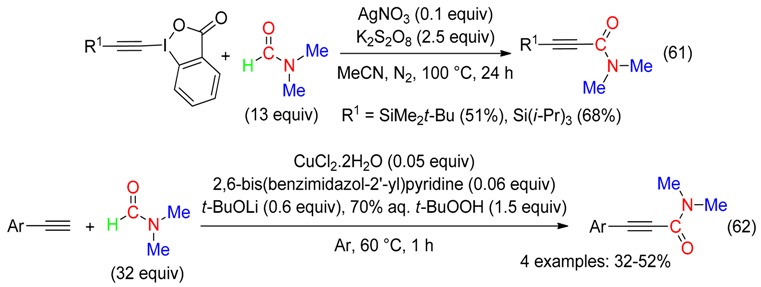


A peroxide was also used for the carbamoylation of 4-arylcoumarin with DMF (Equation (63)) [74].


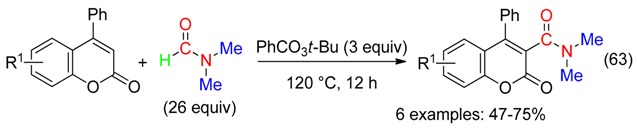


α-Ketoamides were obtained from the domino reaction of toluenes with DMF using (*t*-BuO)_2_, Cs_2_CO_3_ and catalytic amounts of *n*-Bu_4_NI (Equation (64)) [75].


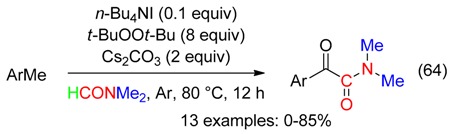


At 100 °C in DMF, Cu catalyst associated to *t*-BuOOH led to unsymmetrical ureas from 2-oxindoles (Equation (65)) [76]. The peroxide would mediate the cleavage reaction, and was the oxygen source of the benzylic carbonyl. That resulted in a ketoamine which undergone the Cu-catalyzed reaction with DMF/*t*-BuOOH, leading to the urea.


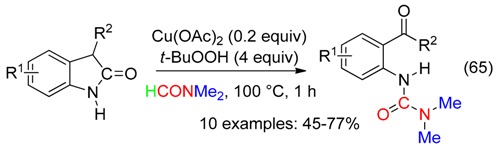


## 10. H Fragment

Semihydrogenation of diaryl alkynes occurred under Ru (Equation (66)) [77] and Pd [78] catalysis with DMF and water as hydrogen source.


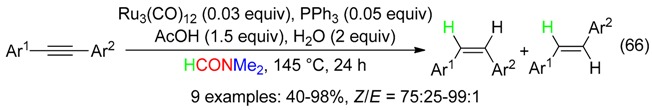


Cobalt porphyrins catalyzed the hydrogenation transfer from DMF to the C(sp^3^)-C(sp^3^) bond of [2.2]paracyclophane (Equation (67)) [79]. DMF was also involved in the Ni-catalyzed intramolecular hydroarylations depicted in Equation (68) [14].


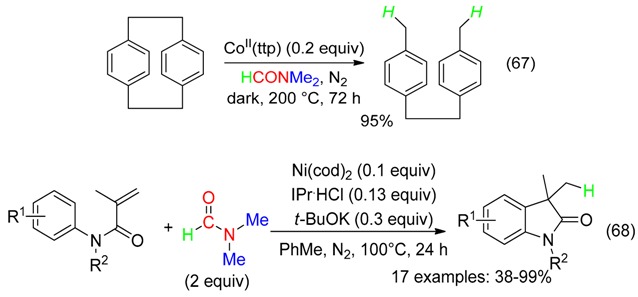


## 11. RC Fragment

New metal-catalyzed and metal-free conditions involving the CH of the formyl group of DMF have been reported for cyclizations leading to heterocycles (Equations (69) [80,81,82] and (70) [83]).


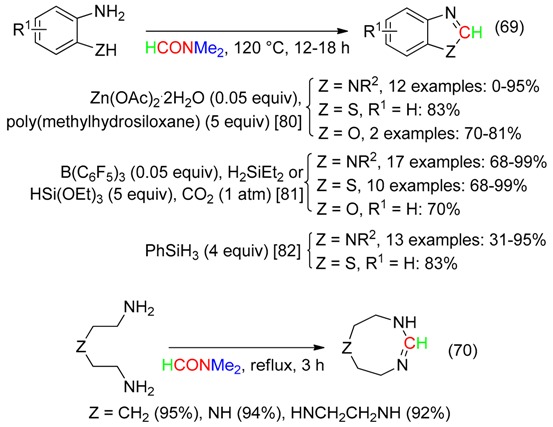


2-Methylbenzimidazoles were obtained from PhSiH_3_-assisted delivery of the CMe of DMAc to benzene-1,2-diamines (Equation (71)) [82].


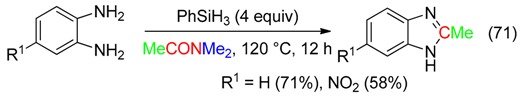


Addition of *p*-tolyllithium to DMF followed by reaction with hydroxylamine hydrochloride afforded 4-methylbenzaldehyde oxime (Equation (72)). The latter underwent cycloaddition with diphenylphosphoryl azide or, in the presence of Oxone^®^, with diethylacetylene dicarboxylate to provide the corresponding 5-aryltetrazole or 3-arylisoxazole, respectively [84].


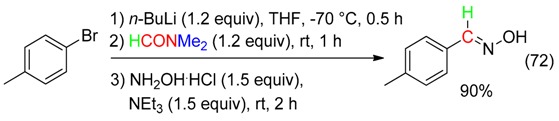


## 12. RCNMe_2_ Fragment

Dihydropyrrolizino[3,2-*b*]indol-10-ones were isolated in fair to high yields from a Cs_2_CO_3_-promoted domino reaction leading to the formation of three bonds with incorporation of the HCNMe of DMF. Such a reaction-type with incorporation of the MeCNMe also occurred in DMAc but with a low yield (Equation (73)) [26].


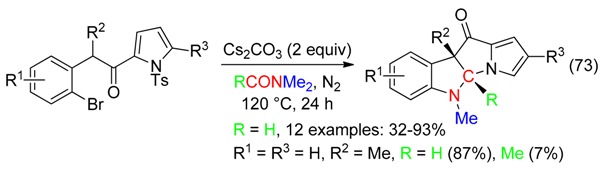


## 13. RC-O Fragment

A 1:1 mixture of CuO and I_2_ led the α-formyloxylation or α-acetoxylation of methylketones by DMF or DMAc, respectively (Equation (74)). α-Iodoketones would be the intermediates as indicated by the reaction of 2-iodo-1-(4-methoxyphenyl)ethanone (Equation (75)). Traces of water delivered the carbonyl oxygen [16].


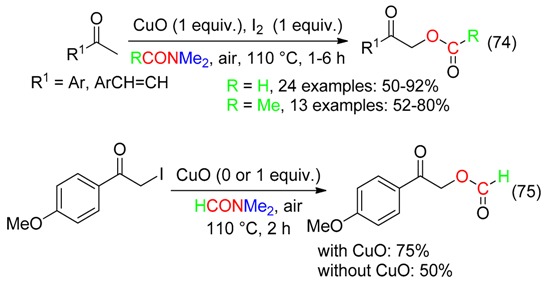


Stereoinversion of the secondary alcohols of a number of carbocyclic substrates was carried out via their triflylation followed by treatment with aqueous DMF (Equation (76)) and subsequent methanolysis [85]. A one pot stereoinversion process was reported.


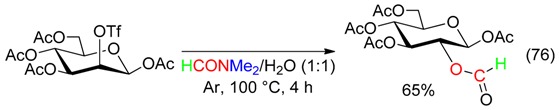


The formyl group of DMF was involved in the triflic anhydride-mediated domino reaction depicted in Equation (77) [86].


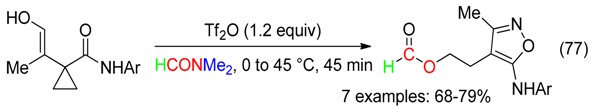


Chloroformyloxylation and chloroacetoxylation of olefinic substrates were performed with PhICl_2_ and either wet DMF or DMAc (Equation (78)) [87]. Styrenes suffered also difunctionalization using aryl diazonium salts and Ru photocatalysis in wet DM (Equation (79)) [28].


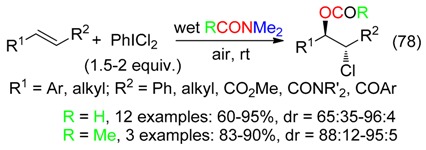



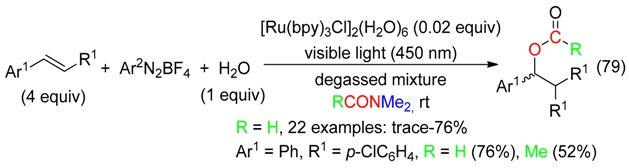


## 14. RC=O Fragment

Metal-catalyzed or CO_2_-mediated C–N and N–H bond metathesis reactions between primary or secondary amines and DM provided the transamidation products, that is formamides or acetamides (Equation (80)) [88,89,90,91].


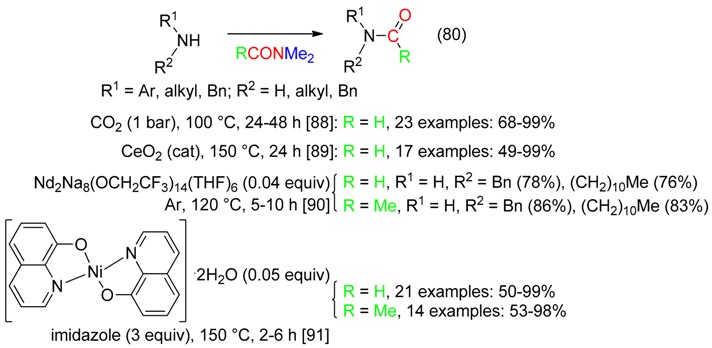


Formylation of aromatic substrates resulted from their treatment with LDA [92] or *n*-BuLi [93] and, subsequently, DMF (Equation (81)).


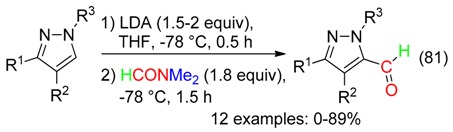


Graphene oxide reacted with DBU and DM to afford, in presence of trace of water, *N*-(3-(2-oxoazepan-1-yl)propyl)formamide or the corresponding acetamide (Equation (82)) [94].


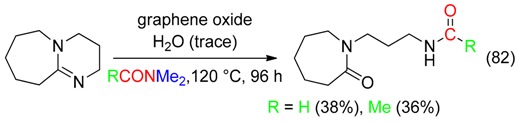


## 15. RC=ON(CH_2_)Me Fragment

*N*-Amidoalkylation of imidazoles and 1,2,3-triazoles with DM effectively arose under various experimental conditions (Equations (83) [95] and (84) [10]).


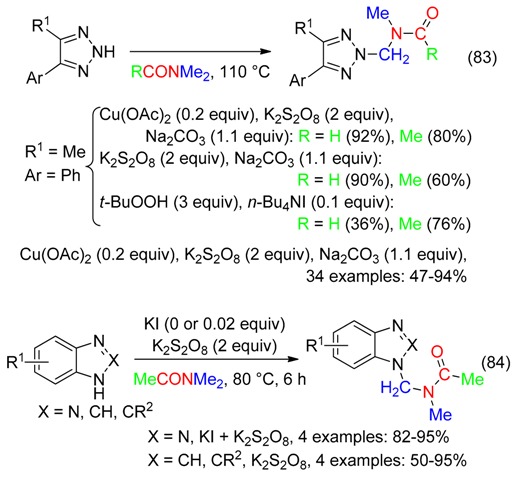


## 16. HC-ONMe_2_ Fragment

Catalysis with Mo(CO)_6_ of the reduction of DMF with triethylsilane afforded a siloxymethylamine (Equation (85)), which was used as a Mannich reagent [96].


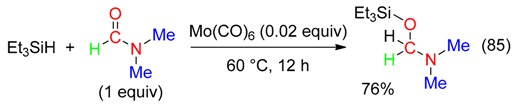


## 17. RC and O Fragment

**Scheme 3:**
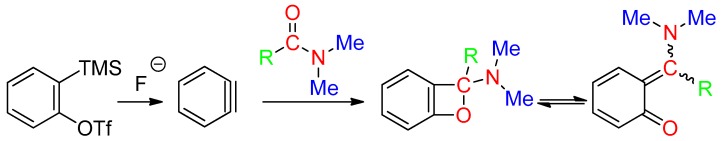
Aryne formation and reaction with DM.

Arynes, which are easily obtained from, for example 2-(trimethylsilyl)phenyl trifluoromethane-sulfonate, undergone a [2 + 2] cyclization with DM giving a benzoxetene and its isomer, the *ortho*-quinone methide (Scheme 3). Trapping of these intermediates provides various products, which contain the formyl or acetyl CH part and the O atom of DM (Equations (86) [19], (87) [20], (88) [21] and (89) [97]), or the HCNMe_2_ and O fragments of DMF (see Section 18).


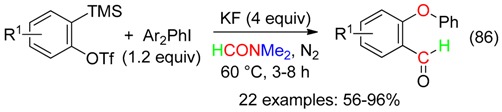



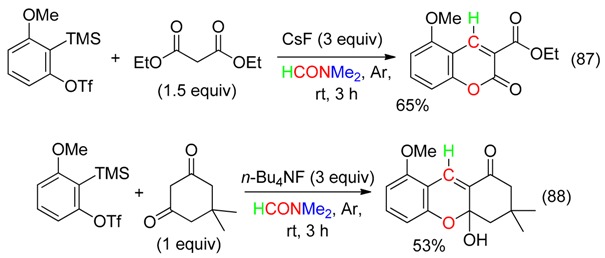


At 115 °C in wet toluene, the hexadehydro-Diels–Alder of tetraynes depicted in Equation (89) was in-situ followed by [2 + 2] cycloaddition reaction with DM leading to multifunctionalized salicylaldehydes and salicylketones [97].


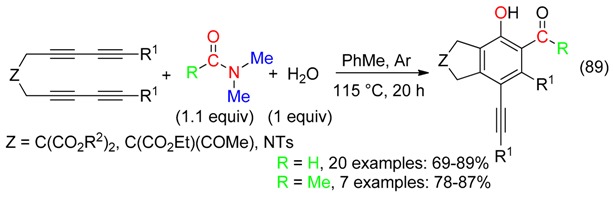


## 18. RCNMe_2_ and O Fragment

All atoms of DMF were inserted in the polyfunctionalized compounds produced from domino reactions involving formation of arynes, cycloaddition with DMF and subsequent trapping with α-chloro β-diesters (Equation (90)) [22], aroyl cyanides (Equation (91)) [23] or diesters of acetylenedicarboxylic acid (Equation (92)) [24].


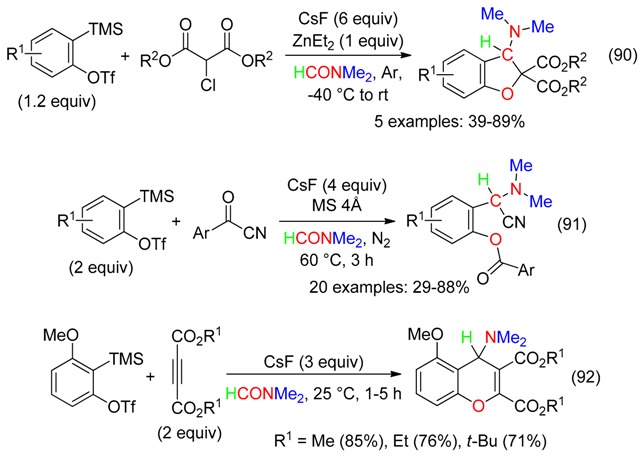


## 19. HC=O and HC Fragment

Lithium thioanisole biscarbanion reacted with two molecules of DMF to afford benzo[*b*]thiophene-2-carbaldehyde (Equation (93)) [98].


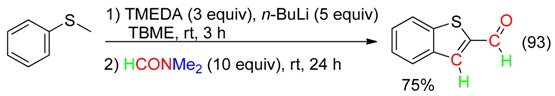


## 20. H and NMe_2_ Fragment

The reaction of DMF with sodium and subsequent addition of terminal activated alkynes afforded the corresponding hydroamination compounds (Equation (94)) [99].


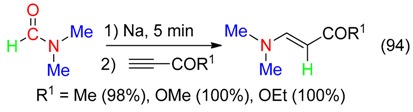


## 21. H and C=ONMe_2_ Fragment

Semicarbazides have been synthetized from additions, mediated with (*t*-BuO)_2_ and catalytic amounts of both NaI and PhCOCl, of the H and CONMe_2_ moieties of DMF to the extremities of the N=N bond of azoarenes (Equation (95)) [100]. The role of NaI and PhCOCl is not clear and, furthermore, exchange of NaI for imidazole led to formylhydrazines (Equation (96)) [100]. The corresponding acetylhydrazine was not formed in DMAc (Equation (96)). The (*t*-BuO)_2_/NaI/PhCOCl/DMF system led to the addition of H and CONMe_2_ to the N=C bond of *N*-benzylideneaniline but with low yield (Equation (97)) [100].


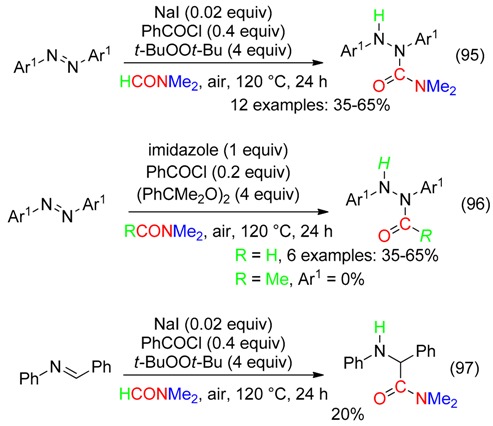


The Ru-catalyzed hydrocarbamoylative cyclization of 1,6-diynes proceeded in DMF to afford cyclic α,β,δ,γ-unsaturated amides (Equation (98)) [101,102,103].


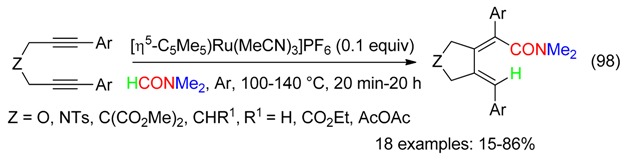


## 22. H, C=ONMe_2_ and NMe_2_ Fragment

Re_2_(CO)_8_[μ-η^2^-C(H)=C(H)Bu](μ-H) undergone reaction with DMF leading to hexenyl/CONMe_2_ and CO/HNMe_2_ exchange of ligands (Equation (99)) [104].


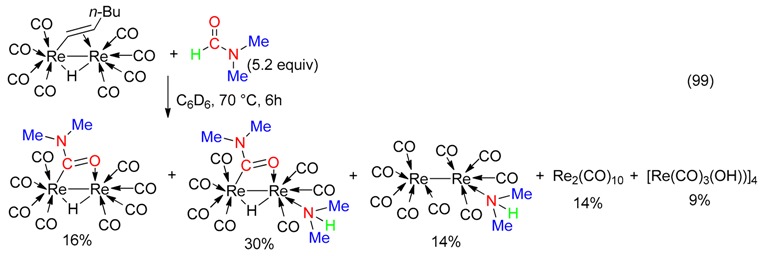


## 23. C=ONMe_2_ and CH Fragment

Couplings between amidines, styrenes and fragments of two molecules of DMF in the presence of *t*-BuOOH and a Pd^II^ catalyst provided pyrimidine carboxamides (Equation (100)) [105]. DMAc may also be the CH source as exemplified with the formation of the *N*,*N*-diethyl-2,4-diphenylpyrimidine-5-carboxamide when *N*,*N*-diethylformamide was the source of the amide moiety (Equation (101)).


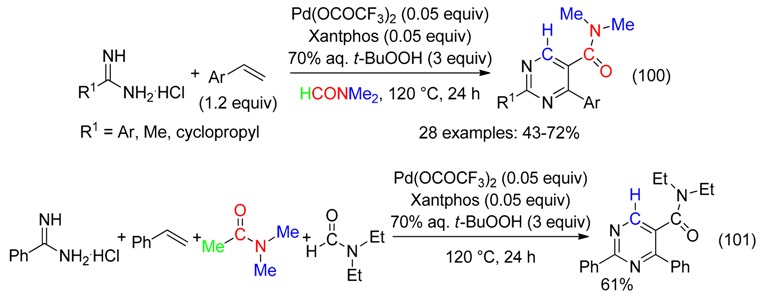


## 24. Reducing or Stabilizing Agent

DMF is a powerful reducing agent of metal salts, hence its use for the preparation of metal colloids [106]. In wet DMF, PdCl_2_ led to carbamic acid and Pd(0) nanoparticles (Equation (102)) [107]. The latter have been associated with the metal-organic framework Cu_2_(BDC)_2_(DABCO) (BDC = 1,4-benzenedicarboxylate), leading to a catalytic system with high activity and recyclability for the aerobic oxidation of benzyl alcohols to aldehydes [108] and Suzuki-Miyaura cross-coupling reactions [107].


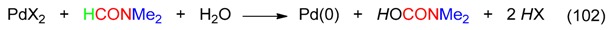


In addition, DMF can act as stabilizing agent of metal colloids to afford effective and recyclable catalysts, based for examples:

- on iron for the hydrosilylation of alkenes (Equation (103)) [109],
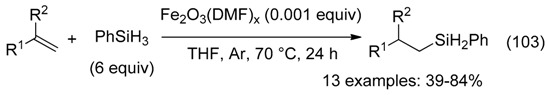
- on palladium for the synthesis of 2,3-disubstituted indoles from 2-halooanilines and alkynes (Equation (104)) [110],
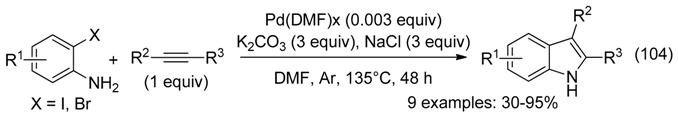
- on copper for Sonogashira–Hagihara cross-coupling reactions (Equation (105)) [111],
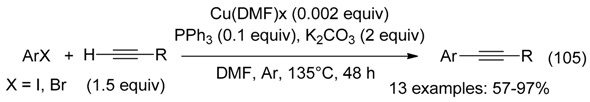
- on iridium for methylation of alcohols (Equation (106)) and amines (Equation (107)), using methanol as the C1 source [112].
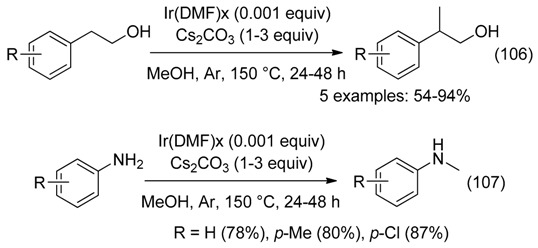

Thermal decomposition of DMF leads to CO, which reacts with water under CuFe_2_O_4_ catalysis to produce hydrogen [113]. In the presence of 2-nitroanilines, this water gas shift reaction was part of a domino reaction involving the reduction of the nitro group followed by cyclisation into benzimidazoles using a CH from the NMe_2_ of DMF (Equation (108)) [113]. Such cyclisation is above documented under different experimental conditions (Equation (18)) [50].


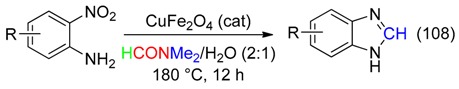


## 25. Conclusions

This minireview highlights recent uses of DMF and DMAc as sources of building blocks in various reactions of the organic synthesis. We assume that new uses of these multipurpose reagents will be reported.
